# Sleep quality and sex modify the relationships between trait energy and fatigue on state energy and fatigue

**DOI:** 10.1371/journal.pone.0227511

**Published:** 2020-01-08

**Authors:** Matthew Manierre, Erica Jansen, Ali Boolani

**Affiliations:** 1 Department of Humanities and Social Sciences, Clarkson University, Potsdam, New York, United States of America; 2 Department of Nutritional Sciences, University of Michigan School of Public Health, Ann Arbor, Michigan, United States of America; 3 Department of Neurology, University of Michigan, Ann Arbor, Michigan, United States of America; 4 Department of Physical Therapy, Clarkson University, Potsdam, New York, United States of America; 5 Department of Biology, Clarkson University, Potsdam, New York, United States of America; Middlesex University, UNITED KINGDOM

## Abstract

The objective of this study was to identify the associations between trait energy and fatigue with state energy fatigue, as well as exploring if these relationships interacted with sex and/or sleep quality. The study population included a convenience sample of adults and college students (n ranges from 687 to 694). Key measures were state and trait mental and physical energy and fatigue scales, PSQI (a measure of sleep quality), and sex. Multiple regression models included age, polyphenol consumption, POMS scores, physical activity, mental load, and caffeine consumption as covariates. Analyses yielded a strong (r = .65) positive association between each trait and state variable. Overall, several statistically significant interactions were identified. First, the relationship between state and trait physical fatigue was particularly strong for women with high trait scores. There were also interactions with sleep quality. In the case of physical fatigue, poor sleep quality magnified the association between physical fatigue trait and state among those with low trait physical fatigue, while sleep quality did not make a difference for those with high trait physical fatigue. Conversely, in the case of physical energy and mental fatigue, good sleep quality was associated with both higher “highs” and lower “lows” of their respective traits; both interactions were present only among males. Our analyses suggest that sleep quality and sex could influence the effects of trait physical and mental energy and fatigue on state. Findings were more complex than initially assumed, suggesting that the interrelationship between trait and state may be modified by how males and females react and adapt to their trait.

## Background

Short-term, transient feelings of fatigue or energy are defined as “state” mood or “state” affect in psychological literature [1–4]; and are related to a number of adverse health and quality of life-related outcomes, including lost work productivity [5], school absences [6], and negative health consequences [7]. Many factors could affect fatigue or energy states. One largely non-modifiable factor that could affect fatigue/energy state is a person’s “trait” affect; the consistent, long-term individual differences in energy or fatigue that are relatively stable over time and context, though gradual changes can occur [8–10]. For instance- feeling mentally exhausted on an “off” day may be reflective of heightened state mental fatigue; however, being constantly mentally drained and unfocused might reflect a trait disposition towards high mental fatigue. These broad, stable traits may arise from hereditary influences, as well as longer-term environmental factors and personality traits [8,9]. In general, trait characteristics are thought to influence both the frequency and intensity of mood states. For example, a person high in trait depression is expected to experience more frequent and intense episodes of state depression in response to stress-provoking situations than those who are low in trait depression [11]. Nonetheless, while the relationships between trait and state moods such as depression [11], anxiety and anger [12,13] have been established, this interplay has not been thoroughly explored in the context of energy and fatigue [8]. In particular, how strongly energy and fatigue trait characteristics predict state energy/fatigue is unknown.

In addition to examining the overall association between trait and state energy and fatigue, we aimed to explore potential interactions. Specifically, is the association between trait and state different between sexes and/or for persons with good versus poor sleep quality? Prior research suggests that sleep might interact with trait in relation to state fatigue/energy. First, feelings of fatigue [14] represent some of the most consistent and noticeable symptoms of short or disrupted sleep. Yet, poor sleep does not impact everyone equally; some end up severely fatigued while others do not notice dramatic changes [15]. Though some of this resilience might be explained by genetic variation [16], another contributor may be a person’s trait energy or fatigue. For example, perhaps those with high trait fatigue notice the effects of poor sleep more keenly, while individuals who have low trait fatigue might prove more resilient to the effects of poor sleep.

The association between trait and state may also differ by sex. Sex differences in the incidence of state fatigue have already been observed, such that women report more feelings of fatigue [17–19]. We could identify no studies that have examined if the interrelationship of trait and state differs between sexes, but there is reason to expect variation within sexes. For example, there may be a stronger association between energy trait and state among women, given that women are typically more cognizant of their typical and current feelings [20]. The associations also might differ in light of the fact that men and women describe their feelings in different ways; for example, across various conditions, women report different symptomology than men even when there are few objective differences [17,21,22]. We therefore hypothesized that there would be sex differences in the relationship between trait and state and, furthermore, there may even be differences in how sleep modifies trait and state according to sex.

In sum, in the present analysis we examined 4 research questions: 1) Are trait energy and fatigue associated with their state counterparts? 2) Are there sex differences in the association between trait and state variables? 3) Does sleep modify the association between trait and state outcomes? And, 4) Are there any interactions between sleep and trait that are sex-specific?

## Methodology

Research was given IRB approval by the Clarkson Institutional Review Board (#Approval# 16–34.1). Respondents provided documented consent on the first page of the web survey. A detailed description of this study methodology has been previously published in Boolani and Manierre [23].

### Data

Subjects were recruited from a small, engineering university and surrounding town (approximate total population 16,000) using the following strategies: 1) announcements made in classes with greater than 50 students through the university; 2) mass emails sent in the fall, spring and summer semesters to students, faculty and staff; 3) flyers posted on campus and in small businesses in the surrounding towns; and 4) through word of mouth. Participants were invited to complete the screening questionnaire administered using SurveyMonkey Inc (San Mateo, California, USA, www.surveymonkey.com). Respondents who were selected for the follow up study were promised $175. Of the 718 respondents who finished the initial screening survey, 30 completed that larger study and received the advertised compensation.

After obtaining Institutional Review Board (IRB) approval (approval# 16–34.1), respondents were administered a survey to determine their eligibility for a study on the effects of caffeine shot consumption on acute changes in mood and cognition. For the purposes of this study there were no eligibility criteria aside from the completion of the survey. After dissemination, 1007 people attempted the survey, but there were 249 incomplete responses and 65 surveys with missing data on study variables. A usable sample of 700 respondents was further reduced to between 670–685 after the removal of outliers, as described in the analysis section. Characteristics of subjects are presented in Table 1. Final variables and Stata syntax for replication can be found publicly on the Open Science Framework via this link: https://osf.io/7svna/?view_only=8e14224607f44474b72ba9cb0ab2e11b (S1 and S2 Files).

**Table 1:** Summary statistics for outcomes and covariates (N = 693).

	Mean/ %	SD	Min	Max
**Primary Variables**				
Female	38.10%		0	1
PSQI>5	51.11%		0	1
Physical energy state^1^	18.19	5.65	7	29
Physical fatigue state^1^	11.67	5.84	3	23
Mental energy state^1^	17.65	5.94	7	29
Mental fatigue state^1^	12.12	6.29	3	25
Physical energy trait	6.57	2.01	3	10
Physical fatigue trait	3.93	1.78	1	7
Mental energy trait	5.94	1.92	3	9
Mental fatigue trait	4.23	1.85	1	7
**Covariates**				
Age>=23 years	82.72%		0	1
Physical Activity	225.10	36.20	168	310
Polyphenol Consumption	96.20	62.68	0	299
Work Day Mental Load	115.95	60.53	8	240
Off Day Mental Load	19.21	14.88	0	56
Caffeine Consumption	12.57	9.85	0	41
POMS Anger	6.67	1.97	5	12
POMS Confusion	5.70	1.74	0	10
POMS Tension	7.79	2.70	5	14
POMS Depression	6.86	2.32	5	13

^1^Note that all four state variables are transformed and/or standardized for final analyses

## Recruitment procedure

### Instruments

#### Sleep

The Pittsburgh Sleep Quality Index (PSQI) was administered to assess sleep quality. This 19-question survey assesses seven underlying dimensions of sleep: Sleep quality, latency, duration, habitual sleep efficiency, sleep disturbance, use of sleep medication and daytime dysfunction over the past month. The scores of the seven dimensions (which each have a range of 0 to 3) are summed to create an overall sleep quality score, with higher scores indicating worse quality sleep (highest possible score = 21). Poor sleep was defined as PSQI score>5 [24].

#### Mental and physical state and trait energy and fatigue scales

In all analyses we considered fatigue and energy separately, since energy and fatigue appear to be separate, albeit correlated constructs [1,3,4,23]. Furthermore, evidence exists that mental and physical forms of energy and fatigue are different but overlapping constructs, so this distinction was also made [23,25]. For example, a person might feel physically energized, or at least not exhausted, but still feel mentally unfocused or drained (i.e. high state physical energy plus high state mental fatigue). To capture this distinction, the mental and physical state and trait energy and fatigue scale was used [26]. The reliability of this scale has been supported by prior analyses [3,3,4,27]. The trait scale is a 12-item measure with 3 items per trait to measure the disposition of mental energy, mental fatigue, physical energy, and physical fatigue. The four trait variables inquired about the frequency of usual feelings, and responses are collected on a 5-point scale ranging from “never” to “always.” Representative statements included: “I feel I have energy” and “I have feelings of being worn out.” The state component had the same 12 items as the trait scale, except that they were measured on a 0 to 10 scale and referred to present state [27]. Among healthy adults, the Cronbach’s alpha coefficients range from 0.82–0.91 [26]. Within the current data, the alpha coefficients ranged from 0.72 to 0.93 (trait mental energy = 0.72, trait mental fatigue = 0.86, trait physical energy = 0.73, trait physical fatigue = 0.82, state mental energy = 0.86, state mental fatigue = 0.93, state physical energy = 0.85, state physical fatigue = 0.91).

Using the background section of the mental and physical state and trait energy and fatigue scale, perceived mental workload and physical activity levels were measured [26]. The Profile of Mood Survey-Short Form (POMS-SF) was used to measure feelings of depression, anxiety, anger, and confusion [28]. Polyphenol and caffeine consumption were measured using surveys that asked subjects about their consumption of different fruits, vegetables, beverages and chocolates [27]. Heightened polyphenol consumption, especially over a long period, has been found to predict moods [29], including trait energy and fatigue [23]. Mental workload, physical activity, depression, anxiety, anger, confusion, polyphenol and caffeine consumption were all controlled for in this analysis. Physical activity was included in models in light of findings that consistent activity is associated with improved feelings of energy and reduced fatigue [30–32]. Details on measures are described in Boolani and Manierre (2019) [23].

### Analyses

#### Preliminary analyses

All variables were first inspected visually and numerically in Stata 14 to identify potential outliers and non-normal distributions. To limit the effect of potential outliers, each variable was winsorized, meaning that extreme scores were replaced with a value that corresponded to an arbitrary percentile of the distribution [33]. Typically, the cut off value was at the 98^th^ or 95^th^ percentile line, or the 2% or 5% line, depending on the direction of the skew. After winsorizing, mental and physical fatigue state variables, the study’s outcomes, they were still heavily skewed to the right. Though regression models are generally robust to violations of normality [34], these two variables were square-root transformed to reduce bias introduced by heteroskedastic and non-normally distributed errors. In addition, all 95% confidence intervals were estimated using robust standard errors. In order to compare the effect sizes across the four dependent variables, each was standardized.

#### Multivariate analyses

Multiple regression models estimated the association between trait scores on state scores, as well as explored their interactions with sleep quality and sex. Each model was carefully checked for outliers using Cook’s D and DFbeta statistics, resulting in the exclusion of between 5 and 23. On average, about 15 cases were removed per model with a standard deviation of 5.15. In addition to this, residuals were examined, and tests for heteroskedasticity, linearity, omitted variable bias, and collinearity were also used.

The first three research questions were answered by a series of four regression models, one for each outcome. Each of these models included interactions between either sex or PSQI with the relevant trait variable. The significance of these interactions was determined both by examining the t-tests of the regression coefficients and a comparison of likelihood ratio tests that compared model fit with and without the interaction terms [35,36]. These tests always agreed, so only the t-test results are shown in text. All models also controlled for age, polyphenol consumption, physical activity scores, caffeine consumption, work and off day mental loads, and POMS scores for anger, confusion, tension, and depression dimensions.

The final research question sought to determine whether any of the previous interactions were sex-specific. Because we were not powered to test three-way interactions, we ran sex-stratified analyses for each of the trait X sleep interactions. Following the suggestions of several authors [37–40], this study presents both unadjusted and Bonferroni adjusted p-values for hypothesis tests. Unadjusted significance tests are included in light of this study’s exploratory nature and concerns that adjustments for multiple comparisons can lead to erroneously heightened type II error rates [37–40]. It can be argued, however, that at least three sets of comparisons are made- one for the overall group, one for males only, and one for females only. To present a more conservative estimate that highlights the possibility of false positives associated with repeated comparisons, a Bonferroni-adjusted significance threshold of p<.016 is also presented on tables, and this cut-off can be applied throughout the manuscript to determine the significance of otherwise unadjusted results. In either case, readers should be mindful of the effect sizes of the results alongside the results of either hypothesis test.

## Results

### Research question 1: Are trait energy and fatigue associated with their state counterparts?

The regression models that inform the first three research questions are summarized here. First, it was found that each trait variable was consistently associated with its respective state counterpart. In simple bivariate analyses, Pearson’s correlation coefficients were between r = .56 and r = .65 (trait-state physical energy, r = .56; trait-state physical fatigue, r = .58; trait-state mental energy, r = .65; trait-state mental fatigue, r = .64). These effects were found to be robust even after controlling for confounders. Because the outcome variable is standardized, regression coefficients are interpreted as the number of standard deviations the outcome is expected to change following a one-unit change in the covariate, which is left in its original scale. Standardized β coefficients reflect how many standard deviations we expect the outcome to change alongside a one standard deviation change in the covariate. The weakest association was for physical fatigue trait on state physical fatigue (b = .272; β = .49; *t* = 10.20; unadjusted p<.001). A similar result was found for physical energy trait where a standard deviation change was associated with a .508 standard deviation increase in state physical energy (b = .251; t = 14.74; unadjusted p<.001).

### Research question 2: Are there sex differences in the association between trait and state variables?

**Fig 1:**
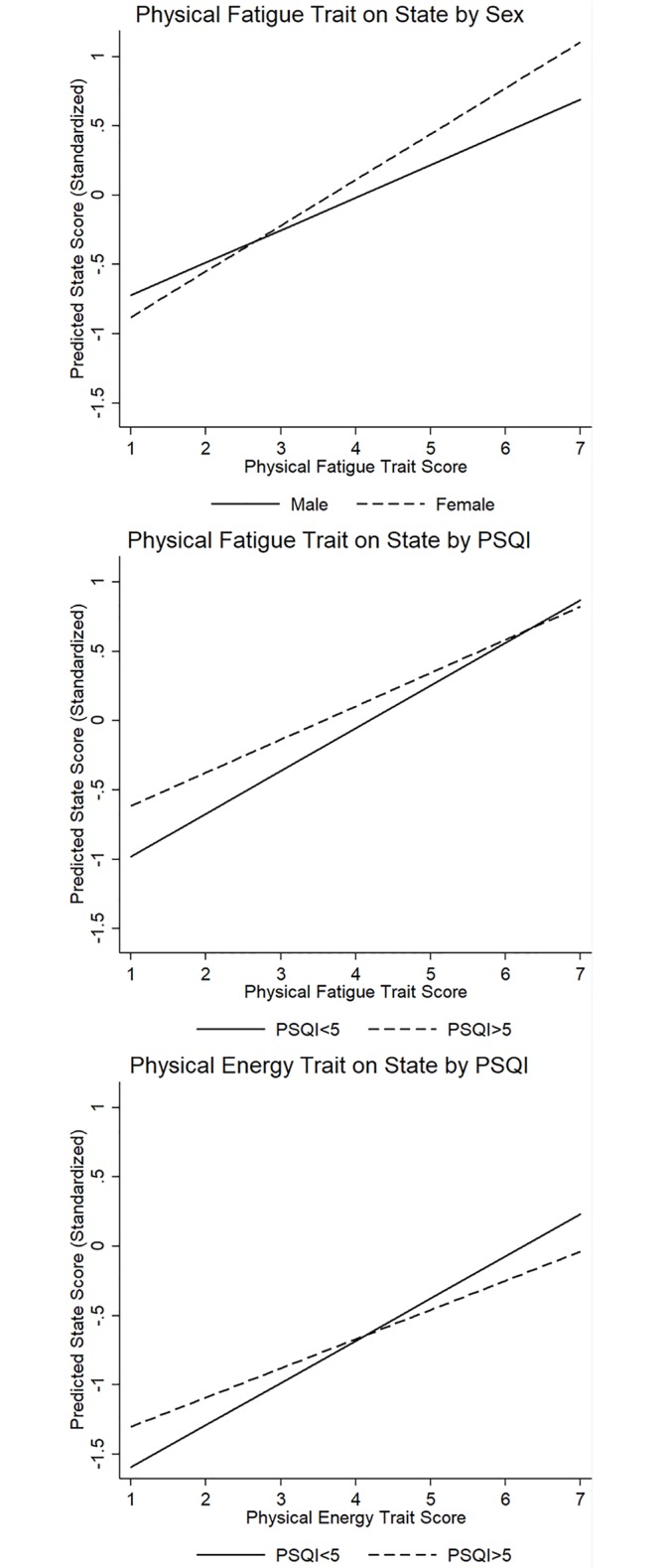
Interactions between trait variables and PSQI or sex in overall models.

Significant interactions for this question and research question 3 are presented graphically (Fig 1), as well as Table 2. Only the interaction between trait physical fatigue and sex was statistically significant (b = .095; β = .218; t = 3.04; unadjusted p = .002). It was found that, though high trait scores were uniformly associated with higher state scores, a female with high trait fatigue was expected to be more fatigued in the present than a similar male. However, at lower trait scores the sex difference vanishes.

**Table 2:** Summary of interactions from overall models^1^.

	Mental fatigue State (N = 688)	Mental Energy State (N = 687)	Physical Fatigue State (N = 694)	Physical Energy State (N = 693)
**No Interactions**				
PSQI >5	0.07	-0.15*†	0.17**†	-0.23***†
Female	0.10	-0.12	0.13*	-0.14*
Trait	0.30***†	0.32***†	0.27***†	0.25***†
**With Interactions**				
PSQI >5	0.26	-0.26	0.43**†	0.38
Female	0.02	-0.22	-0.25	-0.46*
Trait	0.32***†	0.31***†	0.27***†	0.28***†
Female X Trait	0.02	0.02	0.10**†	0.05
PSQI>5 X Trait	-0.05	0.02	-0.07*	-0.09**†

*** unadjusted p<0.001,

** unadjusted p<0.01,

* unadjusted p<0.05,

^†^<p<.05 after Bonferroni adjustment

^1^Controlling for age, polyphenol consumption, physical activity scores, caffeine consumption, work and off day mental loads, and POMS scores for anger, confusion, tension, and depression dimensions

### Research question 3: Does sleep modify the association between trait and state outcomes?

Sleep quality was found to modify both trait physical energy and trait physical fatigue. For physical fatigue, there were stronger associations between trait fatigue and state fatigue among good sleepers than poor sleepers (b = -.684; β = -.175; t = -2.11; unadjusted p = .035). Similarly, there were stronger associations between trait and state physical energy among good sleepers than poor sleepers. association between good sleep quality and higher state physical energy was only present among those with high trait energy (b = -.093; β = -.319; t = -3.08 unadjusted p = .002).

### Research question 4: Is there evidence of sex-specific interactions between sleep and the association between trait and state?

**Fig 2:**
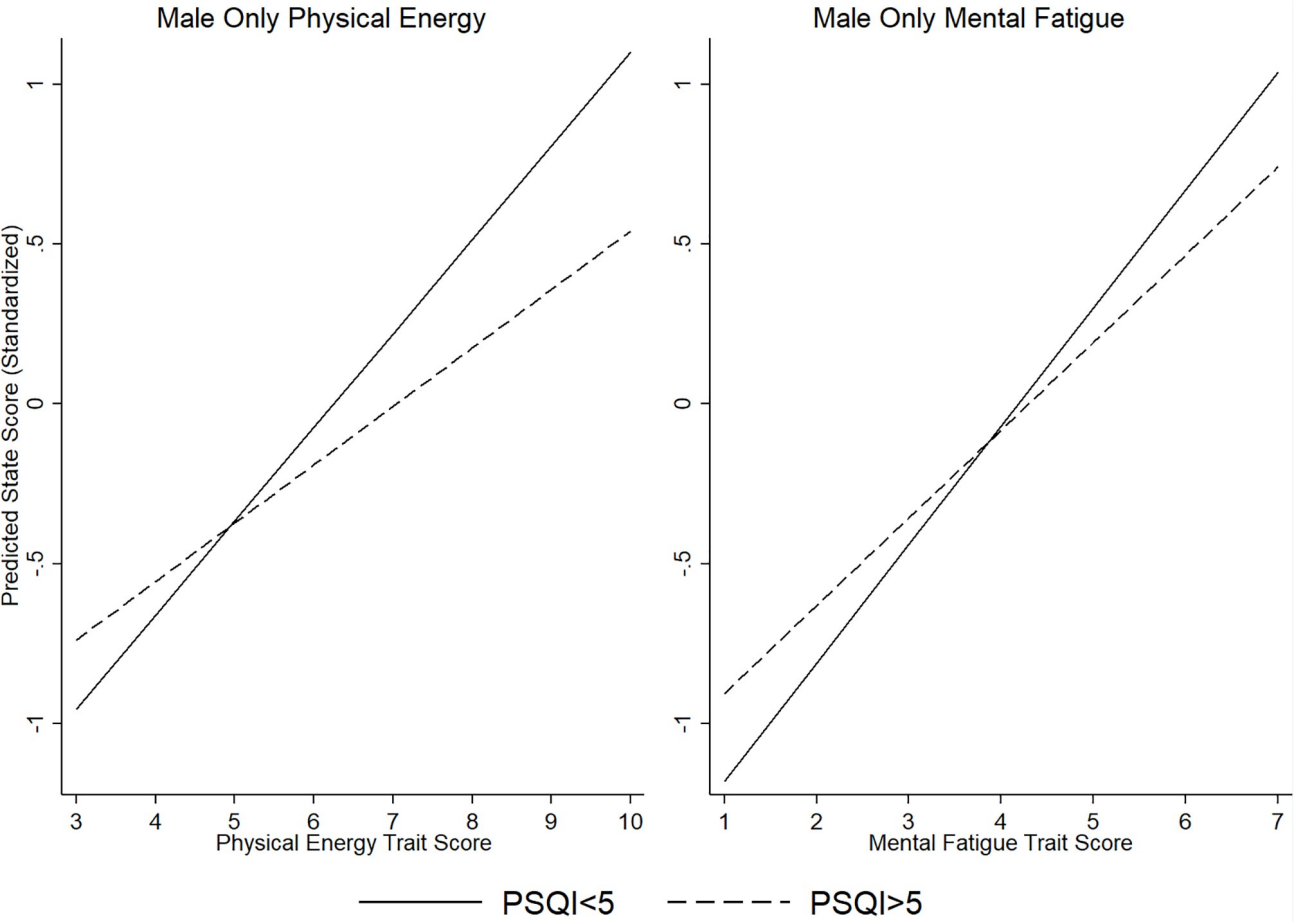
Interactions between sleep and trait in male only model.

Two interactions appeared to only exist in men; as depicted graphically (Fig 2) and in Table 3. First, the aforementioned interaction between sleep and trait physical energy appeared to be driven by men specifically (b = -.110; β = -.397; t = -2.90; unadjusted p = .004). In this case, there was only a lower-magnitude association between trait and state physical energy among men with poor sleep quality compared to good sleep quality. The second sex-specific interaction was for mental fatigue (b = -.095; β = -.272; t = -2.47; unadjusted p = .014), which was not seen in the overall models. The interaction was similar to the previous interaction, in that the association between mental fatigue trait and state was of lower magnitude in the poor sleepers.

**Table 3:** Effects of trait and sleep on state, stratified by sex^1^.

	n	PSQI Above 5	Trait above median	PSQI X Trait
**Males Only**				
State Physical Energy	424	-0.20*†	0.23***†	
State Mental Energy	419	-0.04	0.33***†	
State Physical Fatigue	424	0.17*	0.25***†	
State Mental Fatigue	420	-0.00	0.32***†	
**Females Only**				
State Physical Energy	244	-0.20	0.31***†	
State Mental Energy	249	-0.34***†	0.34***†	
State Physical Fatigue	249	0.11	0.35***†	
State Mental Fatigue	244	0.17	0.32***†	
**Males Only**				
State Physical Energy	424	0.55*	0.29***†	-0.11**†
State Mental Energy	419	0.00	0.33***†	-0.01
State Physical Fatigue	424	0.38*	0.28***†	-0.06
State Mental Fatigue	420	0.37*	0.37***†	-0.10*
**Females Only**				
State Physical Energy	244	0.18	0.34***†	-0.06
State Mental Energy	249	-0.52	0.32***†	0.03
State Physical Fatigue	249	0.44*	0.40***†	0.09
State Mental Fatigue	244	-0.10	0.29***†	0.07

*** p<0.001,

** p<0.01,

* p<0.05,

^†^<p<.05 after Bonferroni adjustment

^1^Controlling for age, polyphenol consumption, physical activity scores, caffeine consumption, work and off day mental loads, and POMS scores for anger, confusion, tension, and depression dimensions

## Discussion

Overall, the aim of this study was to examine the association between trait and state mental and physical energy and fatigue, as well as complicating the relationship by considering the presence of interaction. Our findings suggest that trait mental and physical energy and fatigue are strongly associated with state mental and physical energy and fatigue. In addition, we found evidence of interactions for both sex and sleep, as well as instances where sleep only interacted with trait within males. Though some of these findings are somewhat tentative in light of not passing the conservative Bonferroni-adjusted significance thresholds, these results suggest the possibility that, while the relationship between trait and state is straightforward at first glance, there is also substantial nuance in the relationship to be appreciated.

### Aim 1: Trait predicts state

The finding that trait mental and physical energy and fatigue predicted the complementary state characteristics was in line with expectations set by prior research on state anxiety [12,13,41], anger [13], and depression [11,42]. In each of these cases, and our own, people with low trait characteristics are the most vulnerable groups at any given time to having undesirable state characteristics. As well as identifying an at-risk group, the strong effect of trait on state also suggests that fatigue and energy are not merely ephemeral states- instead, they are rooted in more durable aspects of personality, behavior, and environment. There is also a possibility of reverse causation in the current study, where repeated exposure to state might modify trait. To our knowledge, this is the first study of its kind and further research will need to be conducted to disentangle the array of mediators and moderators that link trait and state energy and fatigue together.

### Aim 2: Sex interacts with trait to predict state

This study initially replicated a result from prior studies [17,18], finding that females in our study had overall higher state and sometimes trait fatigue compared to males (results not shown). Also consistent was that the relationship between trait and state physical fatigue was stronger for women than men. There are several ways to interpret these results, most of which reinforce a narrative that females tend to be disadvantaged with respect to fatigue and energy in numerous ways. Bird and Reiker [43, p.64] propose a multi-layered model of “constrained choice” that describes the interaction of ecological, familial, behavioral, and physiological mechanisms that drive gendered health-related decisions, risk exposures, and biological vulnerabilities. Models such as this provide important context to considering these differences in fatigue scores as manifestations of gendered social arrangements instead of straightforward sex differences. Alongside biological variation, these results may also reflect differences in gendered interpretation of bodily cues/symptomology and their subsequent reporting, reflecting a complex web of socialization and gender performance influences. For instance, enacting masculine traits such as stoicism or toughness might lead to a downplaying of fatigue by men, as has been found in prior studies [44]. Similarly, women are found to more readily communicate symptoms [17–20], which may be reflected here. Environmental differences may also be at play as well, with factors such as gendered division of labor and exposure to institutionalized sexism changing the underlying drivers of both trait and state fatigue. It is not possible to identify the specific web of causation at play in the current study, but subsequent analyses that better account for these factors may yield valuable insights.

### Aim 3 and 4: Sleep quality interacts with trait to predict state, and in a sex-specific manner

We also found that sleep quality modifies the association between trait and state physical energy and fatigue. Specifically, for physical fatigue, there was a much stronger association between physical trait and state fatigue among those with good sleep quality versus those with poor sleep quality. Stated another way, in the presence of poor sleep, physical trait fatigue was less predictive of current physical fatigue state. However, the fact that high trait fatigue predicted higher state fatigue in the presence of good sleep further implies that addressing poor sleep quality may not be sufficient to reduce state fatigue among individuals high in trait fatigue.

The interactions between sleep and physical energy, which turned out to be present only among men, were of a different nature than the interaction between physical fatigue and sleep quality. To illustrate, men who were good sleepers overall had a stronger association between physical energy trait and state than the poor sleepers, including both lower physical energy among those with low trait energy and higher physical energy among those with high trait energy. A similar interaction was observed for mental fatigue. What this might suggest is that those with better sleep quality overall may be more perceptive in describing their feelings of energy, experiencing higher “highs” and lower “lows.” This is supported by studies that have reported poor sleep quality to be associated with poor emotional regulation [45,46]. Another possibility is that some of the “good sleepers” may in fact represent people with excessive sleep duration, which may contribute to feelings of lethargy or fatigue, as well as being a marker for depressive symptomology. This could explain why men with high trait fatigue and good sleep actually have higher predicted scores of mental fatigue than those with high trait fatigue and poor sleep. A third rationalization is that men who are typically fatigued and used to sleeping poorly may have adapted certain gendered behaviors, such as drinking more caffeine or energy drinks, meaning that they are less mentally fatigued in spite of poor sleep. On the other hand, a person who is typically fatigued but sleeps well might not have adopted these behaviors. Although we adjusted for caffeine intake in the analyses, caffeine intake was self-reported and may be subject to reporting errors.

### Limitations

This study has several limitations. Since all data collected were based on retrospective self-report at a single time point, the associations found may be less accurate or informative as compared to the use of multiple assessments and as with all observational data, cannot be viewed as causal. It also depends on a non-random convenience sample with a high dropout rate that is largely restricted to younger adults from a rural northeastern state. Population generalizations should not be made based on these data. Additionally, we measured current state mental and physical energy and fatigue while the sleep quality measures were for a 30-day span. Current mood states may be influenced by immediate events [27,32]. We also opted to use an aggregate measure of sleep quality instead of examining each component of the PSQI. Future research could ascertain which components of sleep quality drive these associations. As already noted, not all results presented met the standard for statistical significance using Bonferroni adjusted cut-offs, raising a degree of ambiguity whether significant results are ignorable or not. Arguably, this only justifies further examining the relationships suggested by this analysis, given that the main reason these results do not meet Bonferroni-corrected significance hinges on the decision to sex-stratify analyses. Lastly, certain relevant measures, such as socioeconomic status, genomic data, alcohol consumption, smoking, and conformity to gender norms were not measured but may have served as confounders or mediators in the current analysis.

## Conclusion

This study reveals a strong linkage between trait energy and fatigue and their respective states. In addition, sex and sleep complicate this relationship in a nuanced manner. Furthermore, there is evidence that at times the moderating effect of sleep is itself sex-specific. Though in general this study affirms the importance of good sleep in optimizing feelings of energy and fatigue, it also identifies new areas for further exploration and identification of causal mechanisms. Future research could elaborate on the current study by using more complex measures of state energy and fatigue that use repeated measurements and varying temporal horizons (e.g. past week, past 30 days). In addition, use of a measure of sleep quality that is sensitive to the possibility of excessive sleep may help to disentangle some of the findings of the current studies. A future study could also use measures such as the conformity to masculine norms inventory [45, 46] to better differentiate between the effects of sex versus gender differences on each outcome. There is also room for research that explores methods for intervention on trait energy and fatigue in both clinical and public health contexts.

## Supporting information

S1 File This Stata file contains all necessary variables for the analysis replication file to be run. (DTA)

S2 File This contains Stata replication syntax that can be used to reproduce all analyses in the paper. (DO)

## References

[1] Loy BD, Cameron MH, O’Connor PJ. Perceived fatigue and energy are independent unipolar states: Supporting evidence. Med Hypotheses. 2018;113:46–51. 10.1016/j.mehy.2018.02.014 29523293 PMC5846196

[2] O’Connor PJ. Evaluation of four highly cited energy and fatigue mood measures. J Psychosom Res. 2004;57(5):435–41. 10.1016/j.jpsychores.2003.12.006 15581646

[3] Boolani A, O’Connor PJ, Reid J, Ma S, Mondal S. Predictors of feelings of energy differ from predictors of fatigue. Fatigue Biomed Health Behav.

[4] Dupree EJ, Goodwin A, Darie CC, Boolani A. A Pilot exploratory Proteomics Investigation of mental fatigue and mental energy In: Advancements of Mass Spectrometry in Biomedical Research. Springer; 2019 p. 601–11. 10.1007/978-3-030-15950-4_36 31347074

[5] Ricci JA, Chee E, Lorandeau AL, Berger J. Fatigue in the US workforce: prevalence and implications for lost productive work time. J Occup Environ Med. 2007;49(1):1–10. 10.1097/01.jom.0000249782.60321.2a 17215708

[6] Bakker RJ, van de Putte EM, Kuis W, Sinnema G. Risk factors for persistent fatigue with significant school absence in children and adolescents. Pediatrics. 2009;124(1):e89–95. 10.1542/peds.2008-1260 19564274

[7] Fukuda S, Yamano E, Joudoi T, Mizuno K, Tanaka M, Kawatani J, et al Effort-reward imbalance for learning is associated with fatigue in school children. Behav Med. 2010;36(2):53–62. 10.1080/08964281003774919 20497943

[8] Watson D. Mood and temperament. Guilford Press; 2000.

[9] Steyer R, Mayer A, Geiser C, Cole DA. A theory of states and traits—Revised. Annu Rev Clin Psychol. 2015;11:71–98. 10.1146/annurev-clinpsy-032813-153719 25062476

[10] Matarán-Peñarrocha GA, Castro-Sánchez AM, García GC, Moreno-Lorenzo C, Carreño TP, Zafra MDO. Influence of craniosacral therapy on anxiety, depression and quality of life in patients with fibromyalgia. Evid Based Complement Alternat Med. 2011;2011. 10.1093/ecam/nep125 PMC3135864 19729492

[11] Cole DA, Nolen-Hoeksema S, Girgus J, Paul G. Stress exposure and stress generation in child and adolescent depression: a latent trait-state-error approach to longitudinal analyses. J Abnorm Psychol. 2006;115(1):40 10.1037/0021-843X.115.1.40 16492094

[12] Spielberger CD, Gorsuch RL. State-trait anxiety inventory (form Y). Consulting Psychologists Press; 1983.

[13] Spielberger CD, Sydeman SJ. State-Trait Anxiety Inventory and State-Trait Anger Expression Inventory. Lawrence Erlbaum Associates, Inc; 1994.

[14] Chervin RD. Sleepiness, fatigue, tiredness, and lack of energy in obstructive sleep apnea. Chest. 2000;118(2):372–9. 10.1378/chest.118.2.372 10936127

[15] Van Dongen HP, Belenky G. Individual differences in vulnerability to sleep loss in the work environment. Ind Health. 2009;47(5):518–26. 10.2486/indhealth.47.518 19834261

[16] Banks S. Behavioral and physiological consequences of sleep restriction. J Clin Sleep Med. 2007;3(05):519–28. 17803017 PMC1978335

[17] Miaskowski C. Gender differences in pain, fatigue, and depression in patients with cancer. JNCI Monogr. 2004;2004(32):139–43. 10.1093/jncimonographs/lgh024 15263057

[18] Schwarz R, Krauss O, Hinz A. Fatigue in the general population. Oncol Res Treat. 2003;26(2):140–4. 10.1159/000069834 12771522

[19] Hunter SK. Sex differences and mechanisms of task-specific muscle fatigue. Exerc Sport Sci Rev. 2009;37(3):113 10.1097/JES.0b013e3181aa63e2 19550202 PMC2909485

[20] Read JG, Gorman BK. Gender and health inequality. Annu Rev Sociol. 2010;36:371–86.

[21] Tang W-R, Yu C-Y, Yeh S-J. Fatigue and its related factors in patients with chronic heart failure. J Clin Nurs. 2010;19(1–2):69–78. 10.1111/j.1365-2702.2009.02959.x 20500245

[22] Fortier-Brochu É, Beaulieu-Bonneau S, Ivers H, Morin CM. Relations between sleep, fatigue, and health-related quality of life in individuals with insomnia. J Psychosom Res. 2010;69(5):475–83. 10.1016/j.jpsychores.2010.05.005 20955867 PMC2958173

[23] Boolani A, Manierre M. An exploratory multivariate study examining correlates of trait mental and physical fatigue and energy. Fatigue Biomed Health Behav.

[24] Buysse DJ, Reynolds CF III, Monk TH, Berman SR, Kupfer DJ. The Pittsburgh Sleep Quality Index: a new instrument for psychiatric practice and research. Psychiatry Res. 1989;28(2):193–213. 10.1016/0165-1781(89)90047-4 2748771

[25] Nozaki S, Tanaka M, Mizuno K, Ataka S, Mizuma H, Tahara T, et al Mental and physical fatigue-related biochemical alterations. Nutrition. 2009;25(1):51–7. 10.1016/j.nut.2008.07.010 18834718

[26] O’Connor P. Mental and physical state and trait energy and fatigue scales. University of Georgia; 2006.

[27] Boolani A, Lindheimer JB, Loy BD, Crozier S, O’Connor PJ. Acute effects of brewed cocoa consumption on attention, motivation to perform cognitive work and feelings of anxiety, energy and fatigue: a randomized, placebo-controlled crossover experiment. BMC Nutr. 2017;3(1):8.

[28] Curran SL, Andrykowski MA, Studts JL. Short form of the Profile of Mood States (POMS-SF): Psychometric information. Psychol Assess. 1995;7(1):80.

[29] Gomez-Pinilla F, Nguyen TT. Natural mood foods: the actions of polyphenols against psychiatric and cognitive disorders. Nutr Neurosci. 2012;15(3):127–33. 10.1179/1476830511Y.0000000035 22334236 PMC3355196

[30] Puetz TW, O’connor PJ, Dishman RK. Effects of chronic exercise on feelings of energy and fatigue: a quantitative synthesis. Psychol Bull. 2006;132(6):866 10.1037/0033-2909.132.6.866 17073524

[31] Dishman RK, Thom NJ, Puetz TW, O’Connor PJ, Clementz BA. Effects of cycling exercise on vigor, fatigue, and electroencephalographic activity among young adults who report persistent fatigue. Psychophysiology. 2010;47(6):1066–74. 10.1111/j.1469-8986.2010.01014.x 20409016

[32] Boolani A, Sur S, Yang D, Avolio A, Goodwin A, Mondal S, et al Six Minutes of Physical Activity Improves Mood in Older Adults: A Pilot Study. J Geriatr Phys Ther 2001 2019; 10.1519/JPT.0000000000000233 31021896

[33] Tukey JW. The future of data analysis. Ann Math Stat. 1962;33(1):1–67.

[34] Lumley T, Diehr P, Emerson S, Chen L. The importance of the normality assumption in large public health data sets. Annu Rev Public Health. 2002;23(1):151–69. 11910059 10.1146/annurev.publhealth.23.100901.140546

[35] Rabe-Hesketh S, Skrondal A. Multilevel and longitudinal modeling using Stata. STATA press; 2008.

[36] Hayes AF. Introduction to mediation, moderation, and conditional process analysis: A regression-based approach. Guilford Publications; 2017.

[37] Althouse AD. Adjust for multiple comparisons? It’s not that simple. Ann Thorac Surg. 2016;101(5):1644–5. 10.1016/j.athoracsur.2015.11.024 27106412

[38] Perneger TV. What’s wrong with Bonferroni adjustments. Bmj. 1998;316(7139):1236–8. 10.1136/bmj.316.7139.1236 9553006 PMC1112991

[39] Rothman KJ. No adjustments are needed for multiple comparisons. Epidemiology. 1990;43–6. 2081237

[40] Feise RJ. Do multiple outcome measures require p-value adjustment? BMC Med Res Methodol. 2002;2(1):8. 12069695 10.1186/1471-2288-2-8 PMC117123

[41] Spielberger CD, Gonzalez-Reigosa F, Martinez-Urrutia A, Natalicio LF, Natalicio DS. The state-trait anxiety inventory. Rev Interam Psicol J Psychol. 2017;5(3 & 4).

[42] Chiappelli J, Nugent KL, Thangavelu K, Searcy K, Hong LE. Assessment of trait and state aspects of depression in schizophrenia. Schizophr Bull. 2013;40(1):132–42. 10.1093/schbul/sbt069 23686021 PMC3885299

[43] Bird CE, Rieker PP. Gender and health: The effects of constrained choices and social policies. Cambridge University Press; 2008.

[44] Courtenay W. Dying to be men: Psychosocial, environmental, and biobehavioral directions in promoting the health of men and boys. Routledge; 2011.

[45] Mauss IB, Troy AS, LeBourgeois MK. Poorer sleep quality is associated with lower emotion-regulation ability in a laboratory paradigm. Cogn Emot. 2013;27(3):567–76. 10.1080/02699931.2012.727783 23025547 PMC3931554

[46] Palmer CA, Alfano CA. Sleep and emotion regulation: an organizing, integrative review. Sleep Med Rev. 2017;31:6–16. 10.1016/j.smrv.2015.12.006 26899742

